# Endoscopic intervention is associated with a lower risk of in-hospital mortality in patients with acute myocardial infarction complicated by gastrointestinal bleeding

**DOI:** 10.3389/fmed.2026.1939304

**Published:** 2026-10-14

**Authors:** Shuwei Li, Yamei Sun, Jie Zhang

**Affiliations:** Department of Gastroenterology, Beijing Anzhen Hospital, Capital Medical University, Beijing, China

**Keywords:** acute myocardial infarction, endoscopic intervention, gastrointestinal bleeding, in-hospital mortality, propensity score matching

## Abstract

**Introduction:**

It remains unclear whether endoscopic intervention affects the prognosis of patients with acute myocardial infarction (AMI) complicated by gastrointestinal bleeding (GIB). This study aimed to evaluate the association between endoscopic intervention and the risk of in-hospital all-cause mortality in patients with AMI complicated by GIB.

**Methods:**

The study had a single-center retrospective cohort design and included consecutive adult patients with AMI complicated by GIB who were hospitalized at Beijing Anzhen Hospital between January 2020 and February 2026. The patients were divided into an endoscopic intervention group and a no-endoscopic intervention group according to whether or not they underwent endoscopic intervention during hospitalization. Baseline differences between the two groups were further addressed using propensity score methods. The primary outcome was in-hospital all-cause mortality. For the primary survival analysis, follow-up began at recognition of GIB, and endoscopic intervention was modeled as a time-varying exposure in Cox regression. Propensity score matching (PSM) was used as a sensitivity analysis to address measured baseline confounding. The secondary outcome was serious in-hospital clinical adverse events after GIB.

**Results:**

A total of 155 hospitalized patients with AMI complicated by GIB were included in the study (endoscopy group, *n* = 57; non-endoscopy group, *n* = 98). In the adjusted time-varying Cox regression model using the recorded intraday times, endoscopic intervention was associated with a lower risk of in-hospital all-cause mortality (adjusted hazard ratio 0.478, 95% confidence interval 0.240–0.951, *P* = 0.035). In the adjusted 24-hour landmark analysis, the association was directionally similar but did not reach statistical significance (hazard ratio 0.433, 95% confidence interval 0.184–1.020, *P* = 0.056). In the propensity-score-matched cohort, endoscopic intervention remained associated with a lower risk of in-hospital all-cause mortality after further adjustment for residual imbalance (adjusted hazard ratio 0.346, 95% confidence interval 0.152–0.786, *P* = 0.011). No statistically significant between-group difference was observed in serious in-hospital clinical adverse events after GIB (odds ratio 1.340, 95% confidence interval 0.660–2.724, *P* = 0.406).

**Discussion:**

In this retrospective cohort, endoscopic intervention was associated with a lower risk of in-hospital all-cause mortality in patients with AMI complicated by GIB, although residual confounding and the uncertainty observed across complementary analyses should be considered when interpreting this association.

## Introduction

Gastrointestinal bleeding (GIB) is a serious in-hospital complication in patients with acute myocardial infarction (AMI) and is associated with a higher risk of mortality and a poor prognosis (1–3). When GIB occurs during hospitalization for AMI, the risk of death may increase markedly (3–5). A study that included 21,135 patients with AMI found that 282 (1.19%) developed GIB during hospitalization, and GIB was associated with a higher risk of in-hospital mortality (odds ratio 2.19) (3). In a nationwide study of 149,477 patients with AMI, those who developed bleeding also had a significantly increased risk of all-cause mortality (hazard ratio [HR] 2.86) (4). Another retrospective study included 6,458 patients with AMI, among whom 131 (2.03%) developed GIB during hospitalization; the in-hospital mortality rate was 11.45% in those with AMI complicated by GIB, which was significantly higher than the rate of 3.56% in those without GIB (5).

For patients with GIB, timely endoscopic intervention can help to identify the source and location of the bleeding and achieve hemostasis using endoscopic clips, topical hemostatic agents, thermal coagulation, or combined approaches (6, 7). In high-risk patients with acute nonvariceal upper GIB, endoscopic intervention can not only identify the bleeding source and treat actively bleeding lesions but also shorten the hospital stay, reduce hospitalization costs, and potentially lower the risk of death (8). However, whether endoscopic intervention can be performed in patients with AMI complicated by GIB remains uncertain. Patients with AMI often have myocardial ischemia, cardiac dysfunction, and hemodynamic instability and are at risk of malignant arrhythmia (9), whereas endoscopic intervention is often performed under sedation or anesthesia. Ischemic heart disease is an independent predictor of periendoscopic cardiac complications (10, 11). During upper gastrointestinal endoscopy, patients with cardiac disease may show fluctuations in cardiopulmonary parameters, including an increased heart rate, an increase in ventricular or supraventricular premature beats, decreased oxygen saturation, and ST-T changes, and patients with coronary artery disease may also develop silent myocardial ischemia during gastroscopy (10). Sedation, procedural stimulation, risk of hypoxia, and volume fluctuations during endoscopy may further increase cardiovascular stress (10, 11). Therefore, clinicians, patients, and family members often decline endoscopic intervention because of concerns that sedation or anesthesia may aggravate hemodynamic instability or induce malignant arrhythmias.

The effect of endoscopic intervention on the prognosis of patients with AMI complicated by GIB remains unclear. Previous studies have focused primarily on patients with acute coronary syndrome and have not further distinguished ST-segment elevation myocardial infarction (STEMI), non-ST-segment elevation myocardial infarction (NSTEMI), and unstable angina (12, 13). The effect of endoscopic intervention on the prognosis remains uncertain in the population with AMI complicated by GIB, which carries both a high bleeding risk and a high cardiovascular risk. Therefore, the aim of this study was to determine the association between endoscopic intervention and in-hospital all-cause mortality in patients with AMI complicated by GIB.

## Materials and methods

### Study population

The study had a single-center retrospective cohort design and was performed at Beijing Anzhen Hospital, a tertiary cardiovascular center. Adult patients who were hospitalized with a diagnosis of AMI complicated by GIB between January 2020 and February 2026 were consecutively screened for enrollment. All included patients had an established diagnosis of AMI at admission, whereas GIB occurred or was recognized at varying times during hospitalization. GIB was defined as the presence of definite clinical signs of bleeding, including melena, hematemesis, hematochezia, or bloody or coffee ground-like fluid from a gastric tube, accompanied by a decrease in hemoglobin of at least 2 g/dL from baseline. Patients with fecal occult blood positivity alone and no other clinical evidence of bleeding were not considered to have GIB. The exclusion criteria were as follows: age <18 years; previous myocardial infarction; missing key variables required for the statistical analyses; and incomplete data that precluded statistical analysis.

### Study groups

The patients were divided into an endoscopic intervention group and a no-endoscopic intervention group according to whether or not they underwent endoscopic intervention during hospitalization. The decision to perform endoscopic intervention was made jointly by gastroenterologists, cardiologists, anesthesiologists, and relevant critical care teams based on the patient's clinical condition with decision-making shared by the patient and family members. Endoscopic intervention in this study refers to therapeutic endoscopy performed after onset of GIB. All endoscopic procedures were performed by experienced gastroenterologists (>10,000 procedures each) and, when necessary, were supported by cardiology, anesthesiology, or critical care teams for perioperative risk management. The procedures were performed in accordance with the British Society of Gastroenterology guidelines for sedation in gastrointestinal endoscopy and the European Society of Gastrointestinal Endoscopy guideline on the diagnosis and management of nonvariceal upper GIB (6, 11).

For patients in the endoscopy group, endoscopy-related data were recorded, including the cause and location of bleeding, the endoscopic hemostatic method used, procedure location, and sedation or anesthesia strategy. Immediate peri-endoscopic adverse events were assessed from the available medical records during the procedure and the immediate post-procedural monitoring period. Sedation and anesthesia strategies were classified based on the medical records as general anesthesia, moderate/deep sedation, moderate/deep sedation with analgesia, or no sedation or anesthesia. Causes of bleeding were classified as ulcer, erosion, Mallory–Weiss syndrome, tumor, and hemorrhoidal/perianal lesions. Ulcers were further classified as gastric, duodenal, complex, or colorectal. Endoscopic hemostatic methods were classified as spraying of a topical hemostatic agent, electrocoagulation, local epinephrine injection, clip placement, or combination hemostasis. Combination hemostasis was defined as simultaneous use of two or more endoscopic hemostatic methods. Bleeding locations were classified as single-site or multiple-site. Single-site bleeding included colorectal, gastric, duodenal, and esophagogastric junction bleeding.

### Outcomes

The primary outcome was in-hospital all-cause mortality. Clinical data for each patient, including vital signs, treatment, and whether patients were receiving aspirin, P2Y12 inhibitors, or anticoagulants before or at the recognition of GIB, were obtained from the electronic medical records. Deaths were confirmed using the electronic medical records and discharge records.

The secondary outcome was serious in-hospital clinical adverse events after GIB, defined as any of the following new-onset events: intra-aortic balloon pump or extracorporeal membrane oxygenation support, acute kidney injury requiring continuous renal replacement therapy, and mechanical ventilation.

### Study design and propensity score methods

To assess baseline differences between the endoscopic intervention and no-endoscopic intervention groups, propensity scores were estimated using a multivariable logistic regression model with receipt of endoscopic intervention as the dependent variable. The propensity score model included age, sex, type of AMI, hemoglobin, blood urea nitrogen, serum creatinine, history of gastrointestinal disease, cardiogenic shock, Killip class III–IV, heart rate and systolic blood pressure at the time of GIB, hypertension, diabetes mellitus, and chronic kidney disease. All covariates were measured before the decision regarding endoscopic intervention. Patients were matched using 1:1 nearest-neighbor PSM without replacement, with a caliper of 0.2 standard deviations of the logit of the propensity score. Exact matching on sex was applied. Among eligible matches within the propensity-score caliper, Mahalanobis distance based on age, hemoglobin, blood urea nitrogen, serum creatinine, heart rate, and systolic blood pressure at the time of GIB was used to further improve covariate balance.

Matching was performed using the MatchIt package in R (14). Covariate balance before and after matching was assessed using standardized mean differences (SMDs) (15, 16), with an absolute SMD of <0.10 used as the target for adequate covariate balance.

The study protocol was approved by the Ethics Committee of Beijing Anzhen Hospital, Capital Medical University (approval No. 2026242x), conducted in accordance with the ethical principles of the Declaration of Helsinki, and reported with reference to the STROBE statement (17). The requirement for informed consent was waived in view of the retrospective observational nature of the research and use of existing clinical data.

### Statistical analysis

Continuous variables are presented as the mean ± standard deviation or median [interquartile range] according to their distribution. Categorical variables are shown as the count (percentage). Continuous variables were compared between groups using the Student's *t*-test or Wilcoxon rank-sum test, and categorical variables were compared using the chi-squared test or Fisher's exact test as appropriate. Covariate balance was assessed using absolute standardized mean differences (SMDs).

The primary outcome analysis was performed in the full cohort using Cox proportional hazards regression with endoscopic intervention modeled as a time-varying exposure. Follow-up began at the recognition of GIB. The recorded clock times of GIB recognition, endoscopic intervention, and death or discharge were used to define exposure and event times. Patients were considered unexposed before endoscopic intervention and exposed thereafter. An unadjusted time-varying Cox model was first constructed. The adjusted model included age, type of AMI, hemoglobin, Killip class, heart rate and systolic blood pressure at the time of bleeding, with sex handled as a stratification variable. Robust variance estimation clustered by patient was used to account for multiple time intervals contributed by individual patients. Hazard ratios (HRs) are reported with 95% confidence intervals (CIs). The proportional hazards assumption for the adjusted time-varying Cox model was assessed using Schoenfeld residuals. A 24-hour landmark analysis was performed as a complementary analysis because most patients who underwent endoscopic intervention received the procedure within 24 h of GIB recognition. Patients who were alive and remained hospitalized 24 h after GIB recognition were included, and exposure status was defined according to whether endoscopic intervention had been performed within the first 24 h. Cox regression was then repeated using the same adjustment variables as in the primary model.

For the secondary outcome of serious in-hospital clinical adverse events after GIB, event rates were compared between the two groups using Fisher's exact test, and odds ratios with 95% CIs were calculated. Endoscopic characteristics were summarized using descriptive statistics.

Discharge alive was considered a competing event for in-hospital death. In the 24-hour landmark cohort, cumulative incidence functions were estimated and compared using Gray's test, and Fine–Gray regression was used to estimate subdistribution hazard ratios. The adjusted Fine–Gray model included endoscopic intervention, age, type of AMI, hemoglobin, Killip class, heart rate and systolic blood pressure at the time of bleeding, and sex.

As an additional sensitivity analysis, time-varying Cox regression was repeated in the propensity-score-matched cohort, with endoscopic intervention modeled as a time-varying exposure. Robust variance estimation clustered by matched pair was used to account for the matched design. To address residual post-matching imbalance, a further model was adjusted for age, type of AMI, hemoglobin, cardiogenic shock, and hypertension. All statistical analyses were performed using R version 4.5.2 (R Foundation for Statistical Computing, Vienna, Austria). Propensity score matching was performed using the MatchIt package. All statistical tests were two-sided, and a *P*-value of <0.05 was considered statistically significant.

## Results

### Study population, baseline characteristics, and endoscopic characteristics

A total of 169 patients with AMI complicated by GIB were screened. After excluding patients aged <18 years, those with previous myocardial infarction, and those with incomplete key data or data that precluded statistical analysis, 155 patients were included in the final cohort. Fifty-seven of these patients underwent endoscopic intervention during hospitalization and 98 did not. The patient screening process is shown in Figure 1. Among all 155 patients, the median interval from admission/AMI diagnosis to GIB recognition was 1 day (IQR, 0–6.5 days; range, 0–66 days). GIB occurred or was recognized on the day of admission in 64 patients (41.3%) and within 1 day of admission in 80 patients (51.6%). The median interval from admission/AMI diagnosis to GIB recognition was 1 day (IQR, 0–7 days) in the endoscopic intervention group and 1 day (IQR, 0–5 days) in the no-endoscopic intervention group.

**Figure 1:**
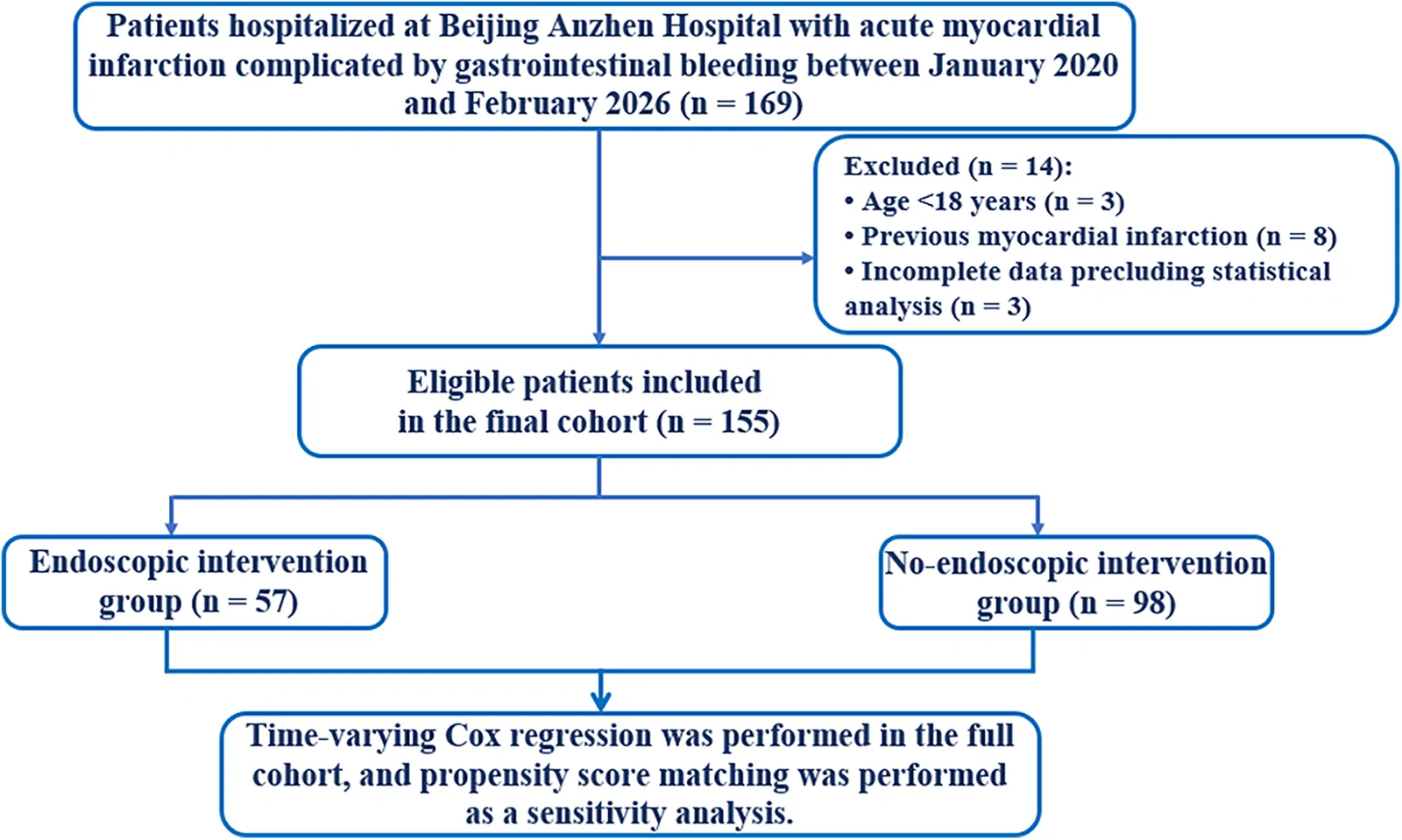
Flow diagram showing the process used to select the study participants.

After 1:1 nearest-neighbor PSM, 43 patients who underwent endoscopic intervention were successfully matched with 43 patients who did not, resulting in a matched cohort of 86 patients. Baseline characteristics before and after matching are shown in Tables 1, 2, respectively. Covariate balance was improved after matching. Most covariates achieved an absolute SMD <0.10; residual imbalance remained for age (SMD 0.124), AMI type (SMD 0.188), cardiogenic shock (SMD 0.155), hemoglobin (SMD 0.161), and hypertension (SMD 0.141). No covariate had an absolute SMD ≥0.20. Covariate balance before and after matching is shown in Figure 2. At the time of GIB recognition, vasopressor support was present in 29.8% (17/57) of patients in the endoscopic intervention group and 27.6% (27/98) in the no-endoscopic intervention group (*P* = 0.854). Mechanical ventilation at GIB recognition was present in 29.8% (17/57) and 23.5% (23/98), respectively (*P* = 0.447). Before or at GIB recognition, aspirin use was 28.1% (16/57) in the endoscopic intervention group and 25.5% (25/98) in the no-endoscopic intervention group (*P* = 0.728). P2Y12 inhibitor use was 22.8% (13/57) and 20.4% (20/98), respectively (*P* = 0.725), and anticoagulant use was 3.5% (2/57) and 2.0% (2/98), respectively (*P* = 0.625). PCI during hospitalization was performed in 42.1% (24/57) of patients in the endoscopic intervention group and 39.8% (39/98) in the no-endoscopic intervention group (*P* = 0.778). Blood transfusion was more frequent in the endoscopic intervention group than in the no-endoscopic intervention group (66.7% [38/57] vs. 37.8% [37/98], *P* < 0.001). Among the 57 patients who underwent endoscopic intervention, the median interval from GIB recognition to endoscopy was 3.8 h (IQR, 2.1–5.6 h; range, 0–88.7 h). Fifty-three patients (93.0%) underwent endoscopy on the same calendar day as GIB recognition, and 55 (96.5%) underwent endoscopy within 24 h of GIB recognition. Esophagogastroduodenoscopy was performed in 45 patients (78.9%) and colonoscopy in 12 (21.1%). All 57 patients underwent therapeutic endoscopic hemostasis. The procedures were performed in the endoscopy unit in 32 patients (56.1%), in the intensive care unit in 11 (19.3%), at the bedside in 10 (17.5%), and in the operating room in 4 (7.0%). Regarding sedation and anesthesia, general anesthesia was used in 34 patients (59.6%), moderate/deep sedation in 10 (17.5%), moderate/deep sedation with analgesia in 1 (1.8%), and no sedation or anesthesia in 12 (21.1%). No immediate peri-endoscopic adverse events were documented in the available medical records during the procedure or the immediate post-procedural monitoring period among the 57 patients undergoing endoscopic intervention. In the endoscopy group (*n* = 57), the most common cause of bleeding was ulceration, which occurred in 40 patients (70.2%) and included gastric ulcer in 15 (26.3%), duodenal ulcer in 11 (19.3%), complex ulcer in 6 (10.5%), and colorectal ulcer in 8 (14.0%). The next most common causes were erosion (*n* = 7, 12.3%) and Mallory–Weiss syndrome (*n* = 5, 8.8%), followed by tumors (*n* = 3, 5.3%) and hemorrhoidal/perianal lesions (*n* = 2, 3.5%). The main hemostatic method was combination hemostasis, which was used in 44 patients (77.2%), followed by topical hemostatic agent spraying in 12 (21.1%) and electrocoagulation in 1 (1.8%). In the endoscopy group (*n* = 57), single-site bleeding occurred in 33 patients (57.9%), including colorectal bleeding in 12 (21.1%), isolated gastric bleeding in 11 (19.3%), isolated duodenal bleeding in 6 (10.5%), and isolated esophagogastric junction bleeding in 4 (7.0%). Multiple-site bleeding occurred in 24 patients (42.1%) and mainly involved the stomach, duodenum, and esophagogastric junction. The endoscopic characteristics of patients in the endoscopy group are shown in Figure 3.

**Table 1:** Baseline characteristics of the endoscopy and non-endoscopy groups before propensity score matching.

Variable	No endoscopy	Endoscopy	*P* value	SMD
Age, years	67.00 [58.25, 72.00]	65.00 [59.00, 70.00]	0.269	0.261
Male sex	75 (76.5%)	46 (80.7%)	0.545	0.102
AMI type	STEMI 49 (50.0%); NSTEMI 49 (50.0%)	STEMI 24 (42.1%); NSTEMI 33 (57.9%)	0.342	0.158
Cardiogenic shock	32 (32.7%)	16 (28.1%)	0.552	0.099
Killip class III–IV	49 (50.0%)	29 (50.9%)	0.916	0.018
History of gastrointestinal bleeding	6 (6.1%)	6 (10.5%)	0.355	0.166
History of gastrointestinal disease	9 (9.2%)	13 (22.8%)	0.019	0.369
Heart rate at bleeding	86.50 [72.00, 100.00]	79.00 [70.00, 100.00]	0.268	0.227
Systolic blood pressure at bleeding	110.00 [98.50, 126.00]	114.00 [90.00, 130.00]	0.929	0.032
Hemoglobin	93.00 [76.25, 126.75]	89.00 [74.00, 118.00]	0.729	0.079
Blood urea nitrogen	8.92 [6.43, 17.64]	11.78 [7.10, 17.99]	0.453	0.084
Serum creatinine	109.30 [80.38, 173.43]	102.00 [77.30, 167.80]	0.569	0.250
Hypertension	68 (69.4%)	33 (57.9%)	0.148	0.236
Diabetes mellitus	47 (48.0%)	31 (54.4%)	0.440	0.129
Chronic kidney disease	20 (20.4%)	6 (10.5%)	0.112	0.287

Values are presented as the median [interquartile range] or number (percentage) as appropriate.

AMI, acute myocardial infarction; NSTEMI, non-ST-segment elevation myocardial infarction; SMD, standardized mean difference; STEMI, ST-segment elevation myocardial infarction.

**Table 2:** Baseline characteristics of the endoscopy and non-endoscopy groups after propensity score matching.

Variable	No endoscopy	Endoscopy	SMD
Age, years	61.00 [56.00, 70.50]	66.00 [60.00, 71.50]	0.124
Male sex	37 (86.0%)	37 (86.0%)	0.000
AMI type	STEMI 24 (55.8%); NSTEMI 19 (44.2%)	STEMI 20 (46.5%); NSTEMI 23 (53.5%)	0.188
Cardiogenic shock	14 (32.6%)	11 (25.6%)	0.155
Killip class III–IV	20 (46.5%)	19 (44.2%)	0.047
History of gastrointestinal bleeding	6 (14.0%)	6 (14.0%)	0.011
History of gastrointestinal disease	8 (18.6%)	9 (20.9%)	0.055
Heart rate at bleeding	79.00 [70.00, 92.50]	76.00 [70.00, 98.50]	0.075
Systolic blood pressure at bleeding	110.00 [96.00, 125.00]	115.00 [90.50, 131.00]	0.081
Hemoglobin	90.00 [76.50, 122.00]	94.00 [78.00, 128.50]	0.161
Blood urea nitrogen	8.49 [6.03, 13.82]	10.06 [6.12, 14.93]	0.036
Serum creatinine	93.50 [78.20, 141.10]	101.70 [78.20, 156.10]	0.080
Hypertension	24 (55.8%)	27 (62.8%)	0.141
Diabetes mellitus	21 (48.8%)	20 (46.5%)	0.047
Chronic kidney disease	3 (7.0%)	4 (9.3%)	0.076

Values are presented as the median [interquartile range] or number (percentage) as appropriate.

AMI, acute myocardial infarction; NSTEMI, non-ST-segment elevation myocardial infarction; SMD, standardized mean difference; STEMI, ST-segment elevation myocardial infarction.

**Figure 2:**
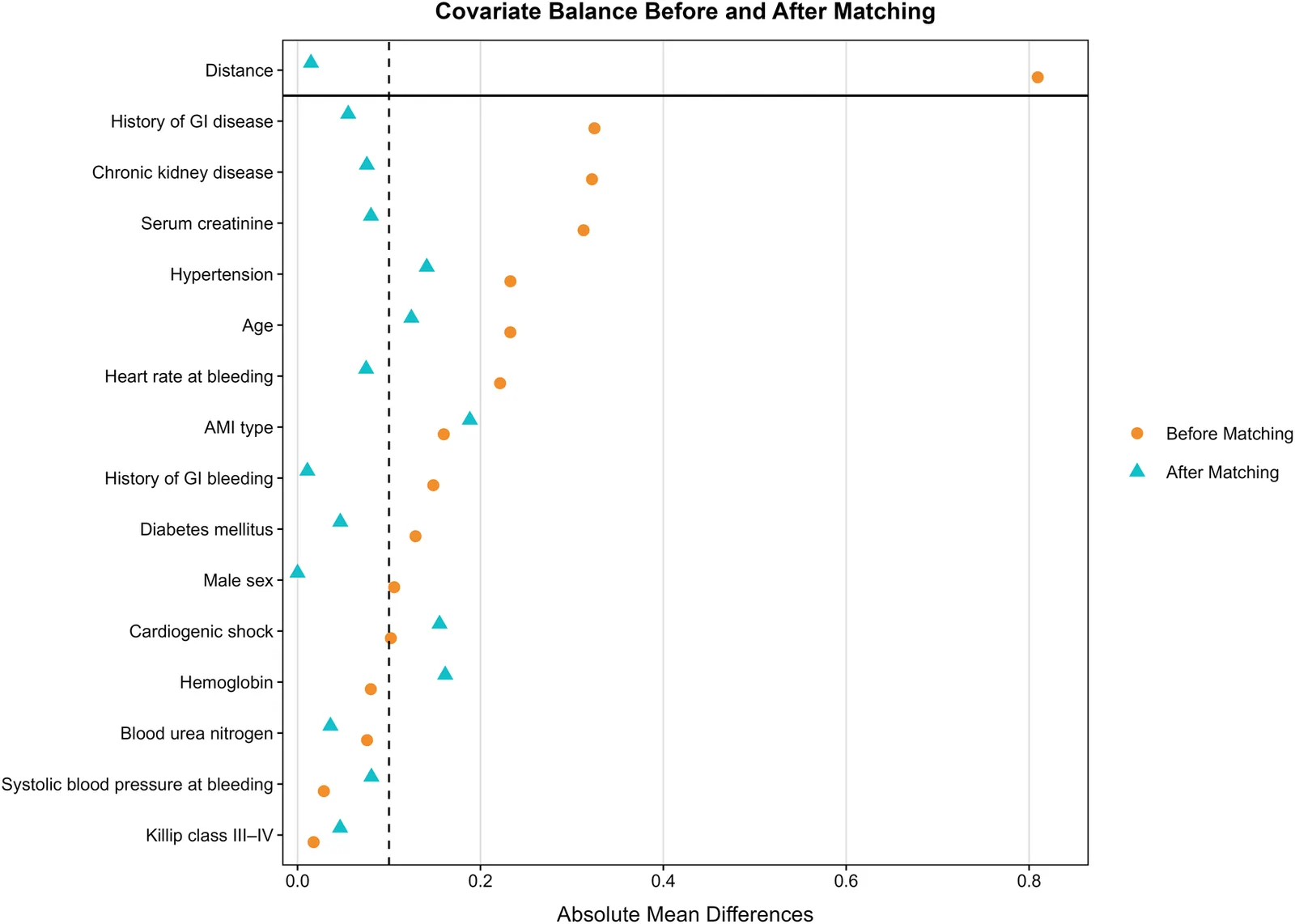
Love plot showing covariate balance before and after propensity score matching. The dashed line represents an absolute standardized mean difference of 0.10.

**Figure 3:**
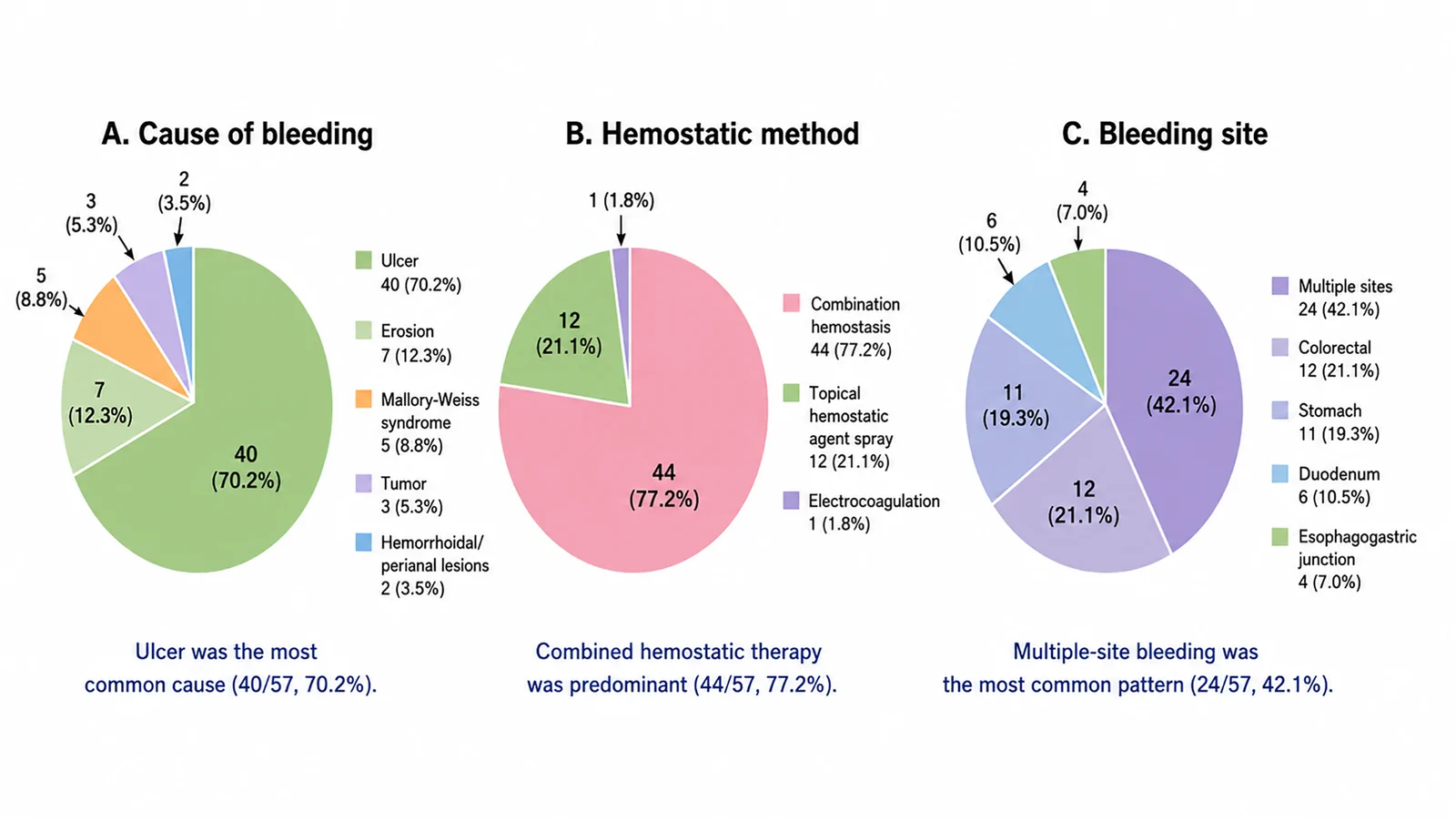
Endoscopic characteristics of patients in the endoscopy group: **(A)** cause of bleeding, **(B)** hemostatic method, and **(C)** bleeding location.

### Primary outcome

In the full cohort, in-hospital all-cause mortality was 17.5% (10/57) in the endoscopic intervention group and 39.8% (39/98) in the no-endoscopic intervention group. Three patients died on the same calendar day as GIB recognition. In the unadjusted time-varying Cox proportional hazards model using recorded intraday times, endoscopic intervention was associated with a lower risk of in-hospital mortality (HR 0.453, 95% CI 0.227–0.907, *P* = 0.025). After adjustment for age, type of AMI, hemoglobin, Killip class, heart rate and systolic blood pressure at the time of bleeding, with sex handled as a stratification variable, the association remained statistically significant (adjusted HR 0.478, 95% CI 0.240–0.951, *P* = 0.035). There was no evidence of violation of the proportional hazards assumption in the adjusted model (global *P* = 0.410).

Complementary analyses showed a generally consistent direction of association. For the 24-hour landmark analysis, 10 patients who died and 5 patients who were discharged alive within 24 h after GIB recognition were excluded, leaving 140 patients who remained alive and hospitalized at the landmark. In this cohort, the unadjusted HR was 0.437 (95% CI 0.200–0.955, *P* = 0.038), and the adjusted HR was 0.433 (95% CI 0.184–1.020, *P* = 0.056). In the complementary competing-risk analysis of the 24-hour landmark cohort, the unadjusted Fine–Gray model yielded an sHR of 0.408 (95% CI 0.190–0.876, *P* = 0.022), and the adjusted model yielded an sHR of 0.398 (95% CI 0.186–0.855, *P* = 0.018).

As a sensitivity analysis, in the propensity-score-matched cohort (43 matched pairs), the time-varying Cox model yielded an HR of 0.313 (95% CI 0.138–0.707, *P* = 0.005). After further adjustment for age, type of AMI, hemoglobin, cardiogenic shock, and hypertension because of residual post-matching imbalance, the adjusted HR was 0.346 (95% CI 0.152–0.786, *P* = 0.011).

### Secondary outcome

In the full cohort, serious in-hospital clinical adverse events after GIB occurred in 49.1% (28/57) of patients in the endoscopic intervention group and 41.8% (41/98) in the no-endoscopic intervention group. Fisher's exact test did not reveal a statistically significant difference between the two groups (OR 1.340, 95% CI 0.660–2.724, *P* = 0.406). Among patients in the endoscopic intervention group, all serious in-hospital clinical adverse events included in this secondary outcome occurred after the endoscopic procedure. No statistically significant between-group differences were observed in the individual components, including IABP, ECMO, CRRT, and mechanical ventilation. The results of the primary and secondary outcome analyses are summarized in Table 3.

**Table 3:** Association between endoscopic intervention and the primary and secondary outcomes.

Model	Effect estimate (95% CI)	*P* value
Primary outcome: in-hospital all-cause mortality		
Unadjusted time-varying Cox model	HR 0.453 (0.227–0.907)	0.025
Adjusted time-varying Cox model	HR 0.478 (0.240–0.951)	0.035
Complementary analyses		
Unadjusted 24-hour landmark Cox model	HR 0.437 (0.200–0.955)	0.038
Adjusted 24-hour landmark Cox model	HR 0.433 (0.184–1.020)	0.056
Unadjusted Fine–Gray model	sHR 0.408 (0.190–0.876)	0.022
Adjusted Fine–Gray model	sHR 0.398 (0.186–0.855)	0.018
Propensity-score-matched sensitivity analyses		
Propensity-score-matched time-varying Cox model	HR 0.313 (0.138–0.707)	0.005
Further adjusted propensity-score-matched time-varying Cox model	HR 0.346 (0.152–0.786)	0.011
Secondary outcome: serious in-hospital clinical adverse events after GIB		
Fisher's exact test	OR 1.340 (0.660–2.724)	0.406

CI, confidence interval; GIB, gastrointestinal bleeding; HR, hazard ratio; OR, odds ratio; sHR, subdistribution hazard ratio.

## Discussion

Based on a single-center cohort of patients with AMI complicated by GIB, this study evaluated the association between endoscopic intervention and in-hospital outcomes using time-varying Cox regression and propensity score methods. In the full cohort, endoscopic intervention was associated with a lower risk of in-hospital all-cause mortality in the adjusted time-varying Cox model using recorded intraday times. The complementary analyses showed a similar direction of association, although the adjusted 24-hour landmark analysis did not reach statistical significance. Taken together, these findings should be interpreted with appropriate caution. No statistically significant difference in serious in-hospital clinical adverse events after GIB was observed between the endoscopic intervention and no-endoscopic intervention groups. GIB can lead to decreased blood volume, anemia, and insufficient tissue perfusion, further aggravating the imbalance between myocardial oxygen supply and demand (18, 19). In patients with preexisting myocardial ischemia or impaired cardiac function, ongoing bleeding may induce or aggravate cardiogenic shock, acute heart failure, malignant arrhythmias, and multiple organ dysfunction, thereby increasing the risk of in-hospital death (19, 20). Successful control of gastrointestinal bleeding may limit ongoing blood loss and improve myocardial oxygen supply, thereby potentially reducing the risk of cardiac death (21, 22). However, because this was a retrospective observational study, these potential mechanisms were not directly evaluated, and a causal relationship cannot be established. Blood transfusion was more frequent in the endoscopic intervention group than in the no-endoscopic intervention group. This difference may reflect differences in bleeding burden and subsequent clinical management; however, the temporal relationship between transfusion and endoscopic intervention could not be reliably determined.

No statistically significant between-group difference was observed in serious in-hospital clinical adverse events after GIB. However, the event rate was numerically higher in the endoscopic intervention group (49.1% vs. 41.8%), and the wide 95% confidence interval for the estimated odds ratio does not exclude a clinically meaningful increase in risk. Previous studies on the safety of endoscopic procedures in patients with acute coronary syndrome or recent AMI have evaluated procedure-related adverse events or post-endoscopic complications, including abnormal vital signs, decreased oxygen saturation, and arrhythmias (23, 24). The present study investigated whether patients with AMI complicated by GIB developed more severe organ dysfunction or required advanced life support after GIB during hospitalization, thereby helping to assess whether endoscopic intervention was associated with in-hospital clinical deterioration in this population. In addition, no immediate peri-endoscopic adverse events were documented in the available medical records during the procedure or the immediate post-procedural monitoring period among patients undergoing endoscopic intervention. This observation should be distinguished from the broader secondary composite outcome, which reflected serious in-hospital clinical deterioration rather than procedure-related complications. In addition, the observation periods for the secondary outcome were not fully comparable between groups because events in the endoscopic intervention group occurred after endoscopy, whereas events in the no-endoscopic intervention group were assessed after GIB recognition. However, the results of this study do not indicate that endoscopic intervention is risk-free. However, the secondary outcome reflects serious in-hospital clinical deterioration rather than procedure-related complications, and the absence of a statistically significant between-group difference should therefore be interpreted cautiously.

This study has several limitations. First, this was a single-center retrospective study and was therefore subject to selection bias and residual confounding. Although propensity score matching was used to account for measured differences between the two groups, unmeasured factors affecting procedural eligibility and treatment selection, as well as residual confounding and confounding by indication, cannot be completely excluded (15, 16). Moreover, propensity score matching was based on eventual receipt of endoscopic intervention rather than risk-set matching at the time of the procedure; therefore, the matched analysis should be interpreted as a sensitivity analysis addressing measured baseline imbalance rather than as eliminating treatment-selection bias. Second, exact clock times of GIB recognition, endoscopic intervention, death, and discharge were retrieved from the medical records and used in the time-to-event analyses to reduce within-day exposure misclassification. Nevertheless, the retrospective nature of timestamp documentation may still have introduced some measurement error. Third, left ventricular ejection fraction was not included in the primary model because of substantial missingness and inconsistent timing of measurement. In addition, the temporal relationship of PCI to GIB and endoscopic intervention and the exact timing of interruption and resumption of antiplatelet or anticoagulant therapy could not be reliably determined in all patients. Blood transfusion may also have occurred after GIB and may have been influenced by bleeding severity and subsequent treatment decisions; therefore, it was reported descriptively rather than treated as a baseline covariate. Fourth, although no immediate peri-endoscopic adverse events were documented in the available medical records during the procedure or the immediate post-procedural monitoring period, the limited sample size and retrospective design preclude definitive conclusions regarding the procedural safety of endoscopy. Finally, the sample size was limited, which reduced the statistical power for evaluating low-frequency adverse events and subgroup differences.

In conclusion, in this retrospective cohort of patients with AMI complicated by GIB, endoscopic intervention was associated with a lower risk of in-hospital all-cause mortality. However, this association should be interpreted in light of residual confounding and the uncertainty observed across complementary analyses. No statistically significant between-group difference in serious in-hospital clinical adverse events after GIB was observed. Successful control of gastrointestinal bleeding may reduce ongoing blood loss and associated hemodynamic stress; however, this potential mechanism was not directly evaluated in the present study. Multicenter, large-sample, prospective studies are needed to further evaluate the association between endoscopic intervention and clinical outcomes in this high-risk population.

## Data Availability

The data analyzed in this study is subject to the following licenses/restrictions: The datasets are not publicly available because they contain potentially sensitive patient information and are subject to institutional privacy and ethical restrictions. Requests to access the datasets should be directed to the corresponding author and will be considered in accordance with applicable regulations and institutional policies. Requests to access these datasets should be directed to JZ, Department of Gastroenterology, Beijing Anzhen Hospital, Capital Medical University, Beijing, China; zhangjie4155@sina.com.
